# Radiological and Clinical Features associated with Epidermal Growth Factor Receptor Mutation Status of Exon 19 and 21 in Lung Adenocarcinoma

**DOI:** 10.1038/s41598-017-00511-2

**Published:** 2017-03-23

**Authors:** Zhang Shi, Xuan Zheng, Ruifeng Shi, Changen Song, Runhong Yang, Qianwen Zhang, Xinrui Wang, Jianping Lu, Yongwei Yu, Qi Liu, Tao Jiang

**Affiliations:** 1 Department of Radiology, Changhai Hospital, Second Military Medical University, Shanghai, China; 2 Clinical Nutrition Department, Changhai Hospital, Second Military Medical University, Shanghai, China; 3 Department of Radiology, Yanan University affiliated hospital, Shanxi, China; 4 Department of Pathology, Changhai Hospital, Second Military Medical University, Shanghai, China

## Abstract

The exon 19 and 21 in Epidermal Growth Factor Receptor (EGFR) mutation are the most common subtype of lung adenocarcinoma, and the strongest predictive biomarker for progression-free survival and tumor response. Although some studies have shown differences in radiological features between cases with and without EFGR mutations, they lacked necessary stratification. This article is to evaluate the association of CT features between the wild type and the subtype (exon 19 and 21) of EGFR mutations in patients with lung adenocarcinoma. Of the 721 finally included patients, 132 were positive for EGFR mutation in exon 19, 140 were positive for EGFR mutation in exon 21, and 449 were EGFR wild type. EGFR mutation in exon 19 was associated with a small-maximum diameter (28.51 ± 14.07) (p < 0.0001); sex (p < 0.0001); pleural retraction (p = 0.0034); and the absence of fibrosis (p < 0.0001), while spiculated margins (p = 0.0095), subsolid density (p < 0.0001) and no smoking (p < 0.0001) were associated with EGFR mutation in exon 21. Receiver Operating Characteristic (ROC) curves suggested that the maximum Area Under the Curve (AUC) was related to the female gender (AUC = 0.636) and the absence of smoking (AUC = 0.681). This study demonstrated the radiological and clinical features could be used to prognosticate EGFR mutation subtypes in exon 19 and 21.

## Introduction

Lung cancer is the leading cause of cancer-related death worldwide^1^, and about 85% lung cancer cases are non-small-cell lung carcinoma (NSCLC), among which lung adenocarcinoma is histologically the most common subtype^2^. Approximately 20% lung adenocarcinoma patients are Epidermal Growth Factor Receptor (EGFR) mutant. EGFR mutation is reported to be as high as 60% in nonsmokers and Asian populations^3^. In the past decade, molecular translational research developments have heralded crucial breakthroughs in the diagnosis and management of lung cancer, particularly the advancement of new targeting therapeutics that are directed against fatal signal pathways involved in cancer growth and progression^4, 5^. The discovery of activating mutations in the tyrosine kinase domain of EGFR promotes the concept of targeted therapy in lung cancer, as the basis for the observed response in patients treated with tyrosine kinase inhibitors (TKIs)^6, 7^. TKIs targeting the EGFR were the first targeted drugs used for the treatment of NSCLC^6^. In addition, patients with EGFR mutations have a higher sensitivity to EGFR TKIs than those with non-EGFR mutations (60–80% *vs.* 10–20%), such as EGFR wild type and unknown mutation status^8^. Clinical trials have clearly demonstrated that patients with EGFR mutations who were treated with targeted TKIs often experienced a longer progression-free survival (PFS) and a higher objective radiographic response rate than patients who used standard first-line chemotherapy^9, 10^. However, according to the classical research^11^, EGFR mutations includes three types (point mutation, multinucleotide in-frame deletion, and in-frame insertion) which have been documented in exon 18 through 21, highlighting that deletion mutation in exon 19 (45%) and point mutation in exon 21 (40–45%) are two most common mutations, accounting for about 90% EGFR mutations in lung adenocarcinoma^4^. It was confirmed for the first time in the IPASS that EGFR mutations in exon 19 and 21 are the strongest predictive biomarkers for PFS and tumor response to first-line gefitinib versus carboplatin/paclitaxel^12^.

Some studies have shown that several clinicopathological factors are associated with a high prevalence of EGFR mutations, such as the female gender, nonsmokers, adenocarcinoma histology, and East Asian origin^13^. Whereas, there are no reliable clinical characteristics that allow for accurate prediction of the EGFR mutation status^14^. For some patients, biopsy samples might be the only tumor materials available for testing the EGFR mutation status and they are often composed of variable ratios of tumor to normal cells^15^. Fortunately, there have been several articles about the relationship between CT features and EGFR mutation status in NSCLC^16–19^. According to a latest report by Rizzo *et al*.^19^, EGFR mutation was significantly correlated with some CT features, such as air bronchogram, pleural retraction, small lesion size, and the absence of fibrosis. What’s more, some researchers thought that if CT-based radiological features associated with the EGFR mutation status could be determined, they could provide a useful clinical predictor in patients with unresectable lung cancer or those whose biopsy is unable to be performed^20^. However, the findings of the relationship between CT features and EGFR mutation status in NSCLC are not consistent with each other. Moreover, none of these studies reported radiological characterization between the subtypes (exon 19 and 21) of EGFR mutation and wild type. In this retrospective study, we performed a radiological analysis to identify some helpful features of EGFR subtype mutation in lung adenocarcinomas in a Chinese cohort of patients.

## Results

**Figure 1:**
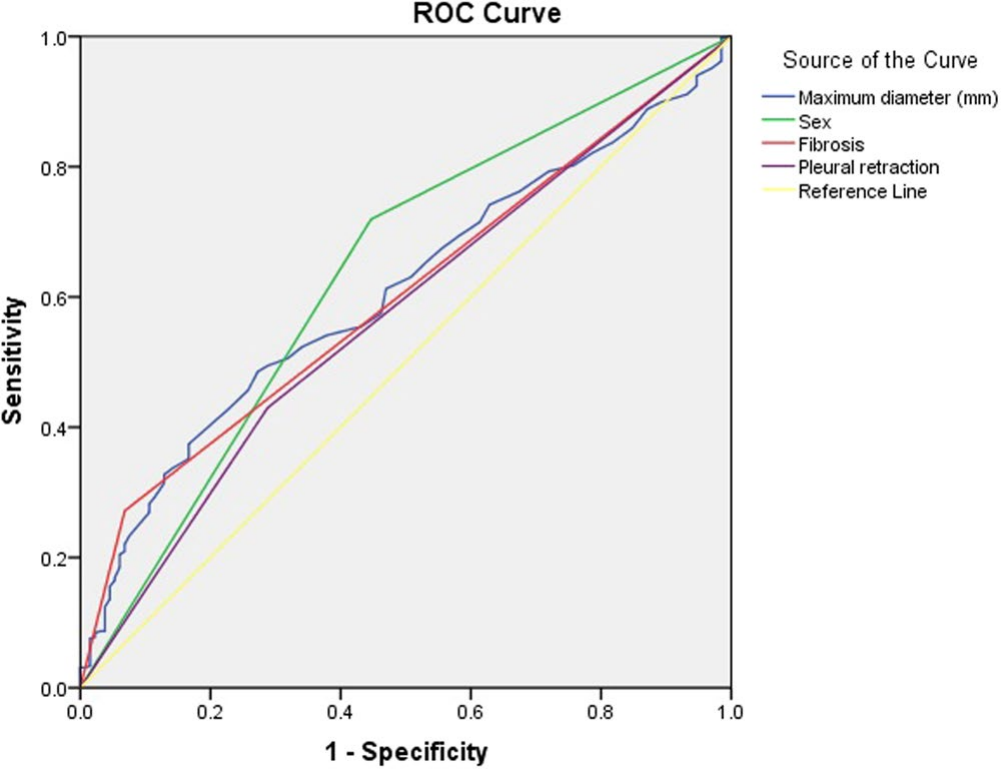
ROC curve for EGFR mutation in exon 19.

According to the inclusion and exclusion criteria, of the 721 included patients, 132 patients (mean age 59.93 ± 9.69 years; M:F = 59:73) exhibited EGFR mutation in exon 19; 140 patients (mean age 60.41 ± 10.04 years; M:F = 56:84) exhibited EGFR mutation in exon 21; and 449 patients (mean age 60.72 ± 10.29 years; M:F = 332:126) exhibited EGFR wild type (Table 1). Table 1 shows that 719 patients underwent contrast-enhanced CT examination. CT and clinical characteristics of EGFR mutations are summarized in Table 2 and Table 3. As shown in Table 2, univariate analysis showed that 11 characteristics, including maximum diameter (28.51 ± 14.07; p < 0.0001); sex (p < 0.0001); ground-glass opacity (p = 0.0055); density (p = 0.0268); vacuole sign (p = 0.0246); necrosis (p = 0.0002); pleural retraction (p = 0.0034); lesion location (p < 0.0001); calcifications (p = 0.0163); fibrosis (p < 0.0001); smoking (p < 0.0001), could be used to help identify EGFR mutation in exon 19. Multiple logistic regression analysis showed that small maximum diameter (odds ratio [OR], 0.982; 95% CI, 0.969–0.995), sex (female) (OR, 0.380; 95% CI, 0.250–0.577), pleural retraction (OR, 2.093; 95% CI, 1.341–3.266), and the absence of fibrosis (OR, 0.288; 95% CI, 0.138–0.600) were important predictors of EGFR mutation in exon 19, where the AUC of ROC was 0.607,0.636,0.602 and 0.571, respectively (Fig. 1).

**Table 1:** CT and clinical characteristics of the study population.

	N/Total (%)
Maximum diameter (mm)*	33.27 (±18.76)
Age (years)*	60.51 (±10.11)
Sex	
Male	438/721 (60.75)
Female	283/721 (39.25)
Lobe	
RUL	209/721 (28.99)
ML	58/721 (8.04)
RLL	133/721 (18.45)
LUL	189/721 (26.21)
LLL	128/721 (17.75)
Mixed	4/721 (0.55)
Shape	
Complex	359/721 (49.79)
Round	201/721 (27.88)
Oval	161/721 (22.33)
Margins Smooth	83/721 (11.51)
Lobulated	660/721 (91.54)
Spiculated/irregular	568/721 (78.78)
Ground-glass opacity	115/721 (15.95)
Density	
Subsolid	112/721 (15.58)
Solid	609/721 (84.42)
vacuole sign	112/721 (15.53)
Cavitation	38/721 (5.27)
Air bronchogram	331/721 (45.91)
Thickening of the adjacent pleura	387/721 (53.68)
Necrosis	298/721 (41.33)
Satellite nodules in primary tumor lobe	251/721 (34.81)
Nodules in non-tumor lobes	333/721 (46.19)
Pleural retraction	448/721 (62.14)
Lesion location	
Central	254/721 (35.23)
Peripheral	467/721 (64.77)
Calcifications	94/721 (13.04)
Emphysema	171/721 (23.72)
Fibrosis	156/721 (21.64)
Pleural contact	471/721 (65.33)
Metastases	70/721 (9.71)
Enlargement of the pulmonary hilar lymph nodes	184/721 (25.52)
Enlargement of the mediastinal lymph node	270/721 (37.45)
contrast enhancement	
15–30 HU	311/721 (43.12)
30–50 HU	204/721 (28.23)
50–70 HU	85/721 (12.24)
>70 HU	62/721 (8.34)
no enhancement	59/721 (8.07)
Smoking	306/721 (42.44)

*Mean (±SD).

**Table 2:** Univariate and multivariate analyses of the EGFR wild type and EGFR mutation in exon 19.

	EGFR			Multivariate Odds Ratio^ (95%CI)
	−N (%)	+N (%)	p-value*	
Maximum diameter^#^	36.08 (±20.57)	28.51 (±14.07)	**<0.0001**	**0.982 (0.969, 0.995)** ^**$**^
Age (years)^#^	60.72 (±10.29)	59.93 (±9.69)	0.4337	
Sex			**<0.0001**	
Male	323 (71.94)	59 (44.70)		**0.380 (0.250, 0.577)**
Female	**126 (28.06)**	**73 (55.30)**		1.00 (Reference)
Lobe			0.2454	
RUL	124 (27.62)	33 (25.00)		
ML	34 (7.57)	14 (10.61)		
RLL	84 (18.71)	32 (24.24)		
LUL	122 (27.17)	26 (19.70)		
LLL	81 (18.04)	27 (20.45)		
Mixed	4 (0.89)	0 (0.00)		
Shape			0.214	
Complex	231 (51.45)	60 (45.45)		
Round	115 (25.61)	44 (33.33)		
Oval	103 (22.94)	28 (21.21)		
Margins Smooth	53 (11.80)	17 (12.88)	0.7388	
Lobulated sign	406 (90.42)	126 (95.45)	0.0675	
Spiculated/irregular margins	338 (75.28)	110 (83.33)	0.0528	
Ground-glass opacity	50 (11.14)	27 (20.45)	**0.0055**	
Density			**0.0268**	
Subsolid	49 (10.94)	26 (19.70)		
Solid	399 (89.06)	107 (80.30)		
vacuole sign	54 (12.03)	26 (19.70)	**0.0246**	
Cavitation	28 (6.25)	6 (4.55)	0.4638	
Air bronchogram	192 (42.76)	65 (49.24)	0.1875	
Thickening of the adjacent pleura	250 (55.68)	67 (50.76)	0.3181	
Necrosis	221 (49.22)	41 (31.06)	**0.0002**	
Satellite nodules in primary tumor lobe	167 (37.19)	45 (34.09)	0.515	
Nodules in non-tumor lobes	202 (44.99)	64 (48.48)	0.4785	
Pleural retraction	256 (57.02)	94 (71.21)	**0.0034**	**2.093 (1.341, 3.266)**
Lesion location			**<0.0001**	
Central	186 (41.43)	30 (22.73)		
Peripheral	263 (58.57)	102 (77.27)		
Calcifications	71 (15.81)	10 (7.58)	**0.0163**	
Emphysema	149 (33.18)	10 (7.58)	1	
Fibrosis	122 (27.17)	9 (6.82)	**<0.0001**	**0.288 (0.138, 0.600)**
Pleural contact	13 (69.71)	81 (61.36)	0.0712	
Metastases	47 (10.47)	13 (9.85)	0.9739	
Enlargement of the pulmonary hilar lymph nodes	132 (29.40)	29 (21.97)	0.0937	
Enlargement of the mediastinal lymph nodes	185 (41.20)	43 (32.58)	0.0744	
contrast enhancement			0.8672	
15–30 HU	187 (41.43)	64 (48.48)		
30–50 HU	128 (28.29)	35 (25.52)		
50–70 HU	55 (11.81)	14 (10.61)		
>70 HU	33 (6.90)	14 (10.61)		
no enhancement	34 (4.22)	12 (4.78)		
Smoking	243 (54.12)	38 (28.79)	**<0.0001**	

CI = Confidence interval.

Note: significant ORs and p-values are in bold.

^#^Mean (±SD).

*Non-parametric two-sample Wilcoxon test for continuous variables.

*Chi-square test and Fisher’s test for categorical variables.

^Obtained by logistic regression model with stepwise selection of variables.

^$^Per 10-mm increase.

**Table 3:** Univariate and multivariate analyses of the EGFR wild type and EGFR mutation in exon 21.

	EGFR	+N (%)	p-value*	Multivariate Odds Ratio^ (95%CI)
	−N (%)			
Maximum diameter^#^	36.08 (±20.57)	28.67 (±14.45)	**<0.0001**	
Age (years)^#^	60.72 (±10.29)	60.41 (±10.04)	0.7526	
Sex			**<0.0001**	
Male	323 (71.94)	56 (40.00)		
Female	126 (28.06)	84 (60.00)		
Lobe			0.1439	
RUL	124 (27.62)	52 (37.14)		
ML	34 (7.57)	10 (7.14)		
RLL	84 (18.71)	17 (12.14)		
LUL	122 (27.17)	41 (29.29)		
LLL	81 (18.04)	20 (14.29)		
Mixed	4 (0.89)	0 (0.00)		
Shape			0.5913	
Complex	231 (51.45)	68 (48.57)		
Round	115 (25.61)	42 (30.00)		
Oval	103 (22.94)	30 (21.43)		
Margins Smooth	53 (11.80)	13 (9.29)	0.4095	
Lobulated sign	406 (90.42)	128 (91.43)	0.7211	
Spiculated/irregular margins	338 (75.28)	120 (85.71)	**0.0095**	**3.330 (1.819, 6.097)**
Ground-glass opacity	50 (11.14)	38 (27.14)	**<0.0001**	
Density			**<0.0001**	**0.304 (0.178, 0.520)**
Subsolid	49 (10.94)	37 (26.43)		
Solid	399 (89.06)	103 (73.57)		
vacuole sign	54 (12.03)	32 (22.86)	**0.0015**	
Cavitation	28 (6.25)	4 (2.86)	0.1224	
Air bronchogram	192 (42.76)	74 (52.86)	**0.0361**	
Thickening of the adjacent pleura	250 (55.68)	70 (50.00)	0.2389	
Necrosis	221 (49.22)	36 (25.71)	1	
Satellite nodules in primary tumor lobe	167 (37.19)	39 (27.86)	**0.0431**	
Nodules in non-tumor lobes	202 (44.99)	67 (47.86)	0.5519	
Pleural retraction	256 (57.02)	98 (70.00)	**0.0062**	
Lesion location			**0.0024**	
Central	186 (41.43)	38 (27.14)		
Peripheral	263 (58.57)	102 (72.86)		
Calcifications	71 (15.81)	13 (9.29)	0.0538	
Emphysema	149 (33.18)	12 (8.57)	1	
Fibrosis	122 (27.17)	25 (17.86)	**0.0262**	
Pleural contact	313 (69.71)	77 (55.00)	**0.0013**	
Metastases	47 (10.47)	10 (7.14)	0.3104	
Enlargement of the pulmonary hilar lymph nodes	132 (29.40)	23 (16.43)	**0.0023**	
Enlargement of the mediastinal lymph node	185 (41.20)	42 (30.00)	**0.0174**	
contrast enhancement			0.2662	
15–30 HU	187 (41.43)	60 (42.86)		
30–50 HU	128 (28.29)	41 (29.29)		
50–70 HU	55 (11.81)	16 (11.43)		
>70 HU	33 (6.90)	15 (10.71)		
no enhancement	34 (4.22)	13 (5.71)		
Smoking	243 (54.12)	25 (17.86)	**<0.0001**	**0.195 (0.121, 0.316)**

CI = Confidence interval.

Note: significant ORs and p-values are in bold.

^#^Mean (±SD).

*Non-parametric two-sample Wilcoxon test for continuous variables.

*Chi-square test and Fisher’s test for categorical variables.

^Obtained by logistic regression model with stepwise selection of variables.

^$^Per 10-mm increase.

**Figure 2:**
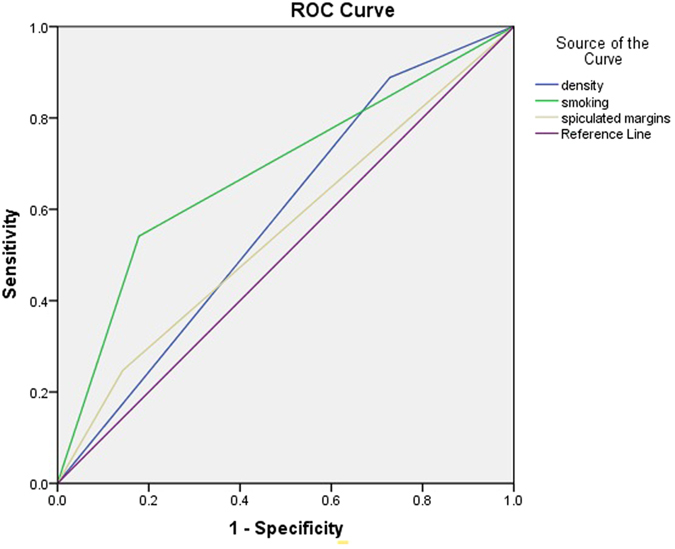
ROC curve for EGFR mutation in exon 21.

As shown in Table 3, of the patients with EGFR mutation in exon 21, there was a significantly higher percentage of maximum diameter (28.67 ± 14.45; p < 0.0001); sex (p < 0.0001); margins (p = 0.0095); GGO (p < 0.0001); density (p < 0.0001); vacuole sign (p = 0.0015); air bronchogram (p = 0.0361); satellite nodules in primary tumor lobe (p = 0.0431); pleural retraction (p = 0.0062); lesion location (p = 0.0024); fibrosis (p = 0.0262); pleural contact (p = 0.0013); pulmonary hilar lymph node enlargement (p = 0.0023); mediastinal lymph node enlargement (p = 0.0174); and smoking (p < 0.0001). Subsequent multivariate analysis confirmed the significance of these features with evidence of three further significant features, which were spiculated margins (OR, 3.330; 95% CI, 1.819–6.097), subsolid density (OR, 0.304; 95% CI, 0.178–0.520), and no smoking (OR, 0.195; 95% CI, 0.121–0.316). Figure 2 shows the ROC curves for the presence of EGFR mutation in exon 21 prediction, and the maximum AUC of the above characteristics was the absence of smoking (AUC = 0.681).

**Figure 3:**
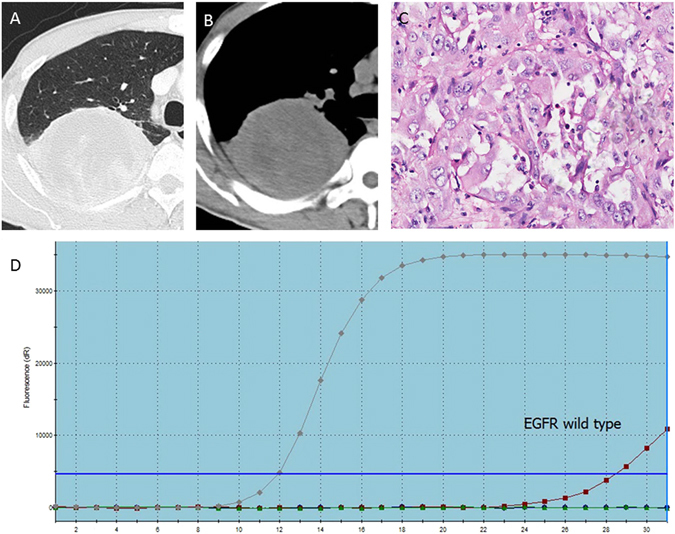
A 46-year-old man with a smoking history of 30 years had right upper lobe lung adenocarcinoma in EGFR wild type, in whom axial CT images (**A,B**) show a solid lump about 90 mm with a litte fibrosis and no pleural retraction or Spicule sign. The PCR picture (**C**) and pathological photo (**D**) show that the red line has no association with the upper line, indicating the EGFR wild type.

**Figure 4:**
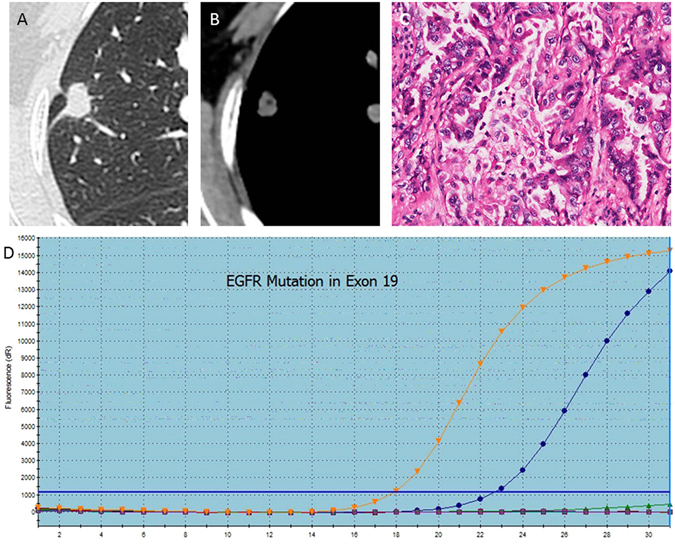
A 62-year-old woman with EGFR mutation of lung adenocarcinoma in the right upper lobe, where the pathological type (**A**) is EGFR mutation in exon 19, and CT images (**B,C**) show a small maximum-diameter lump about 14 mm with obvious pleural retraction and absence of fibrosis. The PCR picture (**D**) shows that the blue line is similar to the upper line, indicating that the subtype of mutation is exon 19 delection.

**Figure 5:**
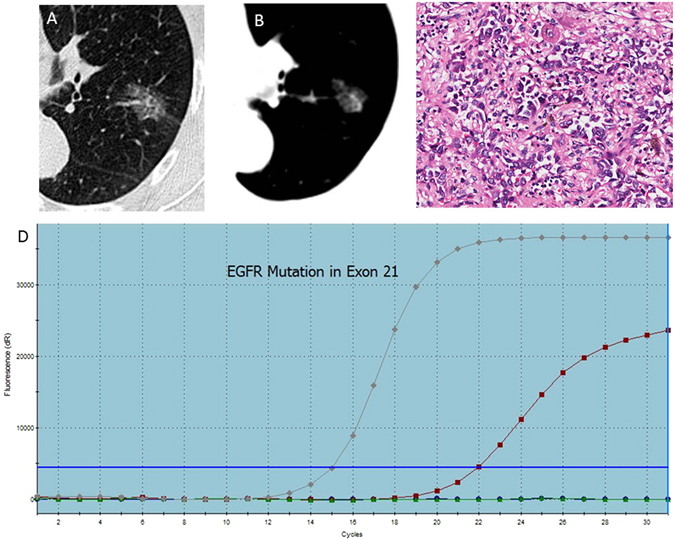
A non-smoking woman with lung adenocarcinoma in the left upper lobe, where PCR images (**A**) and pathological picture (**D**) show that the subtype is exon 21 mutation. CT images (**B,C**) show a subsolid lump with spiculated margins.

According to the above results, there are different CT and clinical features between the EGFR wild type (Fig. 3) and EGFR-mutated subtypes in exon 19 and exon 21. It is clearly known that the female patients with lung adenocarcinoma whose lesions are smaller, less fibrosis and more pleural retraction will have a higher correlativity to the exon 19 mutation (Fig. 4). Similarly, lesions with spiculated margins and subsolid density in non-smoking patients suggested lung cancer with EGFR mutation in exon 21 (Fig. 5).

## Discussion

The results of the present study suggest that the radiological and clinical features could help distinguish the EGFR mutation, especially the subtype of mutation, from the EGFR wild type. Compared with the EGFR wild type, there are some distinct clinicoradiologic characteristics for EGFR mutation of exon 19 (including the female gender, pleural retraction, small lesion diameter, and absence of fibrosis), and non-smoking, spiculated margins and subsolid density for EGFR mutation of exon 21.

The result of previous demographic analysis showed that the female gender, adenocarcinoma histology, the non-smoking status and Asian ethnicity are the most significant factors associated with EGFR mutations and response to EGFR-TKIs^21^, which is somewhat different from the finding of the present study. We found that the female gender was linked with exon 19 mutation, and the non-smoking status was associated with exon 21 mutation.

Although there have been many studies about the radiologic and clinical characteristics in EGFR mutations, all of them only described the clinicoradiological association with the whole types of EGFR mutation without addressing the characteristics of EGFR mutation subtypes. For example, the study by Rizzo *et al*.^19^ pointed out that EGFR mutation was linked with CT features including air bronchogram, pleural retraction, small lesion size, and the absence of fibrosis, in which the last three features are similar to our findings. Recently, Liu *et al*.^20^ found that CT-based radiological features could provide useful information regarding the lung cancer phenotype, and the model that they built could predict the presence of EGFR mutations, where all patients with peripheral lung adenocarcinomas came from Asian. Another study by the same author^22^ reported that CT imaging features of lung adenocarcinomas in combination with clinical variables could be used to better prognosticate the EGFR mutation status than the use of clinical variables alone. Except for the imaging features by CT, there are other imaging modalities to predict EGFR mutations. In recent article by Caicedo *et al*.^23^, they used the PET/CT scans to find that the presence of EGFR mutations did not correlate with ^18^F-FDG uptake. Another study by Stephen *et al*.^24^ indicated that EGFR mutations might drive different metabolic tumor phenotypes that were captured in PET images. Although all the above studies discussed the correlation of the imaging features with the diagnosis of EGFR-mutated lung cancer, none of them reported associations between the radiological features and EGFR mutation subtypes, such as exon 19 mutation.

Classically, EGFR belongs to the ERBB family of cell-surface tyrosine kinase receptors^25^. EGFR is mutated in about 16% tumor specimens from patients with NSCLC^26^. There are several described mutations in the EGFR gene, in which the two most common are short in-frame deletions around the LREA motif of exon 19 (45–50%) and a point mutation (CTG to CGG) in exon 21, resulting in substitution of leucine by arginine at codon 858, L858R (45–50%)^27^. Differences between the subtypes of EGFR-mutated genes result in the discrepancy of the coding protein and the diversity of targeted treatment. The mutations in exon 19 and 21 are responsible for 90% EGFR mutations in lung adenocarcinoma and sensitive to the targeted drugs^28^. Recently, some studies found that there were some differences in treatment and prognosis between exon 19 deletion and exon 21 mutation. A meta-analysis by Zhang *et al*.^29^ indicated that exon 19 deletion might be associated with longer PFS compared to L858 mutation at exon 21 after first-line EGFR-TKIs for patients with NSCLC. Similarly, a report by Sheng *et al*.^30^ suggested that NSCLC patients with EGFR exon 19 deletion had a longer PFS and OS, and a higher response rate after EGFR-TKI therapy compared with those with exon 21 L858R mutation. Therefore, it is very helpful to identify the subtype of EGFR mutation in the clinical treatment of lung cancer.

In this study, we further probed the association between the EGFR wild type and EGFR mutation in exon 19 and 21. Analysis of the radiological features showed that EGFR mutation in exon 19 was associated with a small maximum diameter, pleural retraction and the absence of fibrosis, which similar to the report of Liu *et al*.^22^. Likewise, our study also showed that the two CT features (spiculated margins, subsolid density) and the clinical characteristics (no-smoking) were positive for EGFR mutation in exon 21, which is respectively consistent with the findings of Zhou *et al*.^18^ and Sabri *et al*.^31^. In our opinion, it seems more scientific to analyze the association between the subtypes of EGFR mutation and the wild type than compare all mixed mutated subtypes with the wild type. Of course, this conclusion needs to be confirmed by larger-sample studies. Therefore, we chose 132 EGFR mutations in exon 19 (7.94%) and 140 in exon 21 (8.42%) from more than 1600 cases of lung adenocarcinoma, and found that the subtypes of EGFR mutation could be distinguished by the radiological features, which may prove to be helpful and useful to choose suitable patients and evaluate the clinical treatment. Compared with clinical examinations such as direct sequencing of PCR-amplified genomic DNA, high-resolution melting analysis, fragment analysis, and the amplification refractory mutation system^32^, which are generally expensive and sometimes do not have a high rate of tumor cell detection, radiological features can not only discriminate EGFR-mutated subtypes (exon 19 deletion and exon 21 mutation) but are noninvasive and less expensive, especially for advanced NSCLC patients who cannot receive biopsy^33^. In addition, we tried to find the difference between the exon 19 and 21 mutations and wild type by contrast enhancement, although the difference between them was insignificant (P > 0.05), suggesting that there may be indiscrimination in the angiogenesis and permeability between the wild type and mutation.

There are some limitations in this study. First, this study is retrospective and limited to Chinese populations only. Second, we found that the non-smoking status was more closely correlated with EGFR exon 21 mutation, rather than EGFR mutation in exon 19. It may be attributed to some molecular structures that are sensitive to the tobacco and their changes have effects on the EGFR in the genetic level, while we need more evidences. Third, patients with other EGFR mutation subtypes were not included in our study, and larger patient cohort studies are required to confirm our observation. Fourth, although meaningful imaging features (pleural retraction, spiculated margins, subsolid density, and absence of fibrosis) can indicate the growth way of tumor invasion and the degree of the fibrosis, the current imaging features can’t perfectly reflect the changes of biological conditions. Molecular researches about the relationship between the imaging features and EGFR status can give us more clues in future. Finally, a scoring system should be established from prospective studies in the future, knowing that radiological features obtained from a retrospective study are unable to predict EGFR-mutated subtypes.

In conclusion, this radiologic and clinical analysis of EGFR revealed certain associations between the EGFR wild status and EGFR mutation in exon 19 (including the female gender, pleural retraction, a small lesion diameter and the absence of fibrosis) and exon 21 (non-smoking, spiculated margins and subsolid density). The association of these features may suggest which NSCLC patients are more likely to be EGFR mutation carriers. CT imaging features of lung adenocarcinomas in combination with clinical variables can be used to prognosticate EGFR mutation subtypes.

## Methods

### Patient selection

This study population was retrospectively selected from the patients with NSCLC who underwent EGFR mutation tests between June 2011 and June 2016 in Changhai Hospital (Shanghai, China). The study protocol was approved by the Institutional Review Board of Second Military Medical University (clinical trial registration number: ChiCTR-DOD-15005777). As it was a retrospective research, the committee waived the requirement of informed consent. All experiments were performed in accordance with the approved guidelines. A total of 725 patients from initially retrieved 1662 cases were included in the present study according to the following inclusion criteria^22^: (1) patients with pathologically confirmed diagnosis of NSCLC; (2) patients with preoperative thin-section CT images accessible in our picture archiving and communication system (PACS); (3) patients who underwent EGFR mutation test in our hospital; and (4) patients with complete clinical data including age, sex and smoking history. Of the 725 patients, four patients were finally excluded from the study according to the following exclusion criteria: (1) CT scan performed at another institution or not including the chest at our institution^19^; (2) patients who did not undergo surgery; (3) patients with the EGFR mutation subtype not in exon 19 or 21. Finally, 721 ethnically Chinese patients were reserved for analysis.

### CT image acquisition

CT examinations were randomly performed on two 16-slice Philips CT systems (Philips, Brilliance-16 and MX-16, Netherlands), a 64-slice Siemens system (Siemens, Sensation Cardiac 64, Germany) or a 320-slice CT system (Toshiba, Aquilion ONE, Japan). All examinations were extended in a craniocaudal direction, with or without contrast medium. All images were archived in a digital format. On the two 16-slice CT systems, images were acquired with the following parameters: tube rotation time 0.75 s; pitch 0.938; standard soft-tissue algorithm reconstruction; collimation 24 mm (16 × 1.5 mm); slice thickness 1.0 mm; reconstruction interval 1.0 mm; display field of view (DFOV) 300–360 mm; tube voltage 120 kV; tube current 200 mA/mAs. On the 64-slice CT, images were acquired with the following parameters: tube rotation time 0.5 s; pitch 1.2; standard soft-tissue algorithm reconstruction; collimation 28.8 mm (24 × 1.2 mm); slice thickness 1.0 mm; reconstruction interval 1.0 mm; DFOV 300–410 mm; tube voltage 120 kV; tube current 150 mA/mAs. On the 320-slice CT, images were acquired with the following parameters: tube rotation time 0.5 s; pitch 0.869; standard soft-tissue algorithm reconstruction; collimation 80 mm (160 × 0.5 mm); slice thickness 1.0 mm; reconstruction interval 1.0 mm; display field of view (DFOV) 310–400 mm; tube voltage 120 kV; tube current 300 mA/mAs.

### Assessment of CT features

All qualitative image analyses were performed by three senior radiologists with more than 20-year experience in the diagnostics of thoracic imaging, who were blind to EGFR genomic classification. Discrepancies in interpreting the CT features between them were resolved by discussion until consensus was reached. According to the date of clinical features and CT examinations, each patient was extracted from the medical records. For each patient, the following data from the CT examinations were recorded on an Excel file (Microsoft Office Excel 2013, USA)^19^: (1) maximum diameter (mm) of the lesion detected on the multi-planar reconstructed (MPR) images in a soft tissue window; (2) spot of the lesion, including right upper lobe (RUL), middle lobe (ML), right lower lobe (RLL), left upper lobe (LUL), left lower lobe (LLL), and mixed when the lesion infiltrated more than one lobe; (3) shape, such as complex, round, or oval; (4) margins, indicated as smooth, lobulated, or spiculated/irregular, which was evaluated in the lung window; (5) presence or absence of a ground-glass opacity (GGO); (6) lesion density, indicated as subsolid or solid; (7) lesion with or without vacuole sign; (8)presence or absence of cavitation; (9) presence or absence of air bronchogram; (10) thickening of the adjacent pleura; (11) presence or absence of necrosis in the tumor; (12) presence or absence of satellite nodules in the primary tumor lobe; (13) presence or absence of nodules in non-tumor lobes; (14) presence or absence of pleural retraction; (15) location of the lesion, including central and peripheral; (16) presence or absence of intra-nodular calcifications; (17) presence or absence of emphysema; (18) presence or absence of fibrosis (indicated as presence of honeycombing, traction bronchiectasis, lung architectural distortion, reticulation); (19) presence or absence of pleural contact; (20) presence or absence of metastases (including the intra-pulmonary and distant metastases); (21) presence or absence of pulmonary hilar lymph node enlargement; (22) presence or absence of mediastinal lymph node enlargement; (23) degree of contrast enhancement (indicated as 15–30 HU, 30–50 HU, 50–70 HU, >70 HU, no enhancement).

### Identification of mutations

Tumor specimens for EGFR mutation analysis were obtained from surgical resection. EGFR mutation analyses in two tyrosine kinase domains (exons 19 and 21) frequently seen in lung adenocarcinoma were performed^20^. Tumors were diagnosed as adenocarcinoma and classified according to the 2015 WHO Classification^34^. EGFR-wild type and EGFR-mutated subtypes were determined by an amplification refractory mutation system real-time technology using Human EGFR Gene Mutations Fluorescence Polymerase Chain Reaction (PCR) Diagnostic Kit (Amoy Diagnostics Co., Ltd, Xiamen, China)^22^.

### Statistical analysis

All statistical analyses were performed using SAS software version 9.4 and SPSS 21.0. The mean and standard deviations were expressed for continuous variables (maximum diameter and age), and frequency and percentage for categorical variables in the study population. Univariate analysis was used to assess the association of EGFR wild type and exon 19 and 21 mutations. Non-parametric two-sample Wilcoxon test was used for continuous variables, chi-square test and Fisher’s test for categorical variables, and CMH test for the order variables. Subsequently, multivariate analysis was performed to calculate the odds ratios (OR) with 95% confidence intervals (CI) by a logistic regression model with stepwise selection of variables. As per stepwise selection, effects were entered and removed from the model, so that one or more backward elimination steps could follow each forward selection step. If each forward selection step was significant at the *p* = 0.05 level, the corresponding effect was added to the model. Meanwhile, results of the Wald test for individual parameters were examined at each backward elimination step. The least significant effect not meeting the *p* = 0.05 level was removed. The stepwise selection process terminated when no further effect could be added to the model or when the current model was identical to a previously visited model. Then we corrected the *p*-value for multiple hypothesis testing by SPSS 21.0 with Bonferroni mode and *p*-value < 0.01 are considered statistically significant. Receiver operating characteristic (ROC) curves were drawn for EGFR mutation in exon 19 and 21 according to their significant characteristic, and then the corresponding area under the curve (AUC) was calculated. *P*-values < 0.05 were considered statistically significant.
